# Supplementary material for: A Critical View of the Application of the APEX Software (Aqueous Photochemistry of Environmentally-Occurring Xenobiotics) to Predict Photoreaction Kinetics in Surface Freshwaters
Source: Molecules. 2019 Dec 18;25(1):9. doi: 10.3390/molecules25010009 (PMC7017383; doi:10.3390/molecules25010009)
Supplement: Supplementary file 1 [file molecules-25-00009-s001.zip › 3.2.4_gcc-4.4.0.zip!/3.2.4_gcc-4.4.0/3.2.4_gcc-4.4.0/doc/octave/html/interpreter/Integer-Data-Types.html]

Integer Data Types - Untitled


Next: Bit Manipulations,
Previous: Single Precision Data Types,
Up: Numeric Data Types


---

### 4.4 Integer Data Types

Octave supports integer matrices as an alternative to using double
precision. It is possible to use both signed and unsigned integers
represented by 8, 16, 32, or 64 bits. It should be noted that most
computations require floating point data, meaning that integers will
often change type when involved in numeric computations. For this
reason integers are most often used to store data, and not for
calculations.

In general most integer matrices are created by casting
existing matrices to integers. The following example shows how to cast
a matrix into 32 bit integers.

```
     float = rand (2, 2)
          => float = 0.37569   0.92982
                     0.11962   0.50876
     integer = int32 (float)
          => integer = 0  1
                       0  1
```

As can be seen, floating point values are rounded to the nearest integer
when converted.

— Built-in Function: **isinteger** (x)  
> Return true if x is an integer object (int8, uint8, int16, etc.).
> Note that `isinteger (14)` is false because numeric constants in
> Octave are double precision floating point values.
>
>
>
> **See also:** isreal, isnumeric, class, isa.

— Built-in Function: **int8** (x)  
> Convert x to 8-bit integer type.

— Built-in Function: **uint8** (x)  
> Convert x to unsigned 8-bit integer type.

— Built-in Function: **int16** (x)  
> Convert x to 16-bit integer type.

— Built-in Function: **uint16** (x)  
> Convert x to unsigned 16-bit integer type.

— Built-in Function: **int32** (x)  
> Convert x to 32-bit integer type.

— Built-in Function: **uint32** (x)  
> Convert x to unsigned 32-bit integer type.

— Built-in Function: **int64** (x)  
> Convert x to 64-bit integer type.

— Built-in Function: **uint64** (x)  
> Convert x to unsigned 64-bit integer type.

— Built-in Function: **intmax** (type)  
> Return the largest integer that can be represented in an integer type.
> The variable type can be
>
> `int8`: signed 8-bit integer. `int16`: signed 16-bit integer. `int32`: signed 32-bit integer. `int64`: signed 64-bit integer. `uint8`: unsigned 8-bit integer. `uint16`: unsigned 16-bit integer. `uint32`: unsigned 32-bit integer. `uint64`: unsigned 64-bit integer.
>
> The default for type is `uint32`.
>
>
>
> **See also:** intmin, bitmax.

— Built-in Function: **intmin** (type)  
> Return the smallest integer that can be represented in an integer type.
> The variable type can be
>
> `int8`: signed 8-bit integer. `int16`: signed 16-bit integer. `int32`: signed 32-bit integer. `int64`: signed 64-bit integer. `uint8`: unsigned 8-bit integer. `uint16`: unsigned 16-bit integer. `uint32`: unsigned 32-bit integer. `uint64`: unsigned 64-bit integer.
>
> The default for type is `uint32`.
>
>
>
> **See also:** intmax, bitmax.

— Function File: **intwarning** (action)  
— Function File: **intwarning** (s)  
— Function File: s = **intwarning** (...)  
> Control the state of the warning for integer conversions and math
> operations.
>
> "query": The state of the Octave integer conversion and math warnings is queried. If there is no output argument, then the state is printed. Otherwise it is returned in a structure with the fields "identifier" and "state". ``` intwarning ("query") The state of warning "Octave:int-convert-nan" is "off" The state of warning "Octave:int-convert-non-int-val" is "off" The state of warning "Octave:int-convert-overflow" is "off" The state of warning "Octave:int-math-overflow" is "off" ``` "on": Turn integer conversion and math warnings "on". If there is no output argument, then nothing is printed. Otherwise the original state of the state of the integer conversion and math warnings is returned in a structure array. "off": Turn integer conversion and math warnings "on". If there is no output argument, then nothing is printed. Otherwise the original state of the state of the integer conversion and math warnings is returned in a structure array.
>
> The original state of the integer warnings can be restored by passing
> the structure array returned by `intwarning` to a later call to
> `intwarning`. For example
>
> ```
>           s = intwarning ("off");
>           ...
>           intwarning (s);
> ```
>
>
>
>
> **See also:** warning.

- Integer Arithmetic
